# Paradoxical intention as a treatment for insomnia disorder: study protocol for a mixed-methods pilot trial

**DOI:** 10.1136/bmjopen-2024-086676

**Published:** 2024-10-10

**Authors:** Osame Salim, Markus Jansson-Fröjmark, Christina Sandlund, Annika Norell

**Affiliations:** 1 School of Behavioural, Social and Legal Sciences, Örebro University Faculty of Humanities and Social Sciences, Örebro, Sweden; 2 Department of Clinical Neuroscience, Karolinska Institute & Stockholm Health Care Services, Stockholm, Sweden; 3 Division of Family Medicine and Primary Care, Department of Neurobiology, Care Sciences and Society, Karolinska Institute, Stockholm, Sweden; 4 Academic Primary Health Care Centre, Region Stockholm, Stockholm, Sweden

**Keywords:** sleep medicine, internet, psychosocial intervention, psychiatry

## Abstract

**Introduction:**

Paradoxical intention (PI) is an insomnia treatment developed in the 1970s, which instructs patients to gently attempt to remain awake while in bed at night with the lights off. Previous research indicates PI’s potential in improving insomnia, although no study has been conducted in the last few decades during which the insomnia diagnostic criteria have changed. Additionally, there are knowledge gaps regarding outcomes related to wake after sleep onset, the treatment mechanisms as well as the acceptability and feasibility of the treatment. This study therefore aims to address these gaps by assessing the potential mechanisms, preliminary efficacy, and patient experience and acceptability of PI.

**Methods and analysis:**

We aim to include 40 adult participants with insomnia, aged 18 and above, from the Swedish general population. In this uncontrolled pilot study using a mixed-methods approach, both qualitative and quantitative data will be collected. The trial will be conducted on a self-help online platform, accessible from participants’ homes, with weekly phone call support by therapists. Process and outcome measures will be assessed weekly across a 4-week intervention period and at a 3-month follow-up. A subset of participants will be asked to participate in qualitative semistructured interviews regarding the treatment.

**Ethics and dissemination:**

Ethical approval for this project has been granted by the Swedish Ethical Review Authority (Dnr: 2023-06594-01). All participants will sign informed consent forms on a web service application prior to enrolment. From this mixed-methods study, we anticipate insights into the preliminary efficacy and mechanisms of paradoxical intention for treating insomnia, enriched by patient experience data. Results will be disseminated through peer-reviewed publications. The findings will inform adaptations to the treatment protocol and serve as groundwork for a possible larger scale randomised controlled trial.

**Trial registration number:**

NCT06259682.

**STRENGTHS AND LIMITATIONS OF THIS STUDY**

- The mixed-methods approach of this study strengthens the results by allowing a comprehensive analysis of paradoxical intention’s (PI) efficacy, mechanisms and patient acceptability, informed by qualitative interviews for potential treatment adaptations.
- The inclusion of patients with both singular and comorbid insomnia enhances the generalisability of findings to a broader population, reflecting real-world clinical settings.
- The study’s uncontrolled design limits the ability to isolate the effects of PI from non-specific treatment factors.
- The limited sample size, though typical or even large for a pilot study, may still limit detecting nuanced effects and understanding relationships between process variables.

## Introduction

 Insomnia disorder is characterised by difficulties initiating, returning to or waking up too early from sleep, in combination with daytime consequences (worry or functional impairment). Approximately 10% of the population meets the criteria for insomnia disorder^1^ and the condition is related to several negative effects for those inflicted (eg, psychological distress, decreased quality of life and higher sick leave).^2 3^ Insomnia disorder also tends to remain chronic if untreated^4^ which further highlights the importance of finding and developing effective treatment options for the condition.

During the 1970s, Ascher and Efran^5^ applied paradoxical intention (PI) as an insomnia treatment for patients who had not improved sufficiently after behavioural treatments. PI was described as instructing patients with sleep onset insomnia to try to remain awake for as long as possible rather than to focus on trying to fall asleep as people with sleep issues tend to do. After the first promising study in 1978, other trials in following years also investigated the efficacy of PI with similar results.^6^,^8^ In later years, PI has been reviewed favourably.^9^

Previously, two theoretical models of PI have been presented to account for why PI results in positive effects for patients with insomnia disorder. Originally, PI was based on the understanding that people with insomnia struggle because they believe sleep is a voluntary process and put too much effort into trying to fall asleep.^5^ This effort to sleep was thought to lead to frustration and activate the autonomic nervous system, which makes falling asleep harder. The cycle of self-monitoring, heightened arousal, performance anxiety, sleep effort and failing to fall asleep feeds into itself and creates a vicious cycle. In this view, PI works by reducing performance anxiety around sleep.

In a second model, PI was seen in the light of the attention-intention-effort model (AIE).^10^ This model acknowledges the aspects of anxiety and effort but highlights additional factors. It suggests that people with insomnia pay excessive selective attention to ‘threatening’ sleep-related cues (like a beating heart or external noises), which then leads to a conscious intention to fall asleep. This intention inhibits the normal process of winding down (dearousal), and the heightened intention to sleep leads to both direct (actively trying to sleep) and indirect (extending time in bed) sleep efforts. The AIE model views PI as a way to manipulate this intensified sleep intention by encouraging the individual to remain passively awake or to abandon the direct intention to try to fall asleep.

Additionally, the concept of acceptance, which stems from acceptance and commitment therapy (ACT)^11^ could also be considered a possible mechanism. Indeed, in one study of PI, the instructions given to participants involve ‘giving up trying’^12^ which is similar to instructions seen in later ACT treatments for insomnia.^13^ Some studies have also shown that mindfulness interventions that target psychological flexibility, the theoretical model underlying acceptance and mindfulness interventions, to be effective in treating insomnia disorder.^13 14^ This further gives credence to the idea that acceptance or psychological flexibility is a possible mechanism in insomnia and could be relevant to PI.

Lastly, the concept of metacognition has been increasingly investigated in insomnia during the last decade. Metacognitions have been mentioned in diverse theoretical contexts, which makes synthesis more difficult. For instance, Ong *et al*^15^ developed a metacognitive model of insomnia, which shares mainly connections with other mindfulness and acceptance models. Other researchers have looked at metacognition in the context of the self-regulatory executive function model.^16^ However, these differences withstanding, a core definition of metacognitions might be retained as ‘mental events about one’s thinking’.^17^ Certain studies show that metacognitions regarding insomnia have a mediating role between emotional stability and the effects of self-reported sleep quality.^18^ Even trait neuroticism has been shown to be partially mediated by metacognitive assumptions in the domain of insomnia.^19^ This line of research taken together shows that metacognitive theories are emerging as promising in explaining phenomena within insomnia. PI can be seen as challenging the underlying metacognitions about need for control and danger of unrestrained cognition that may be involved in insomnia disorder.

### Prior evidence of PI for insomnia

The efficacy of PI has been reviewed over the years. The American Academy of Sleep Medicine has twice reviewed PI and concluded that the treatment component is an empirically supported intervention^20^ and categorised PI as a well-established treatment.^21^ Between 2006 and 2018, articles on PI for insomnia are non-existent which reflects its diminishing use in clinical practice. A narrative review on cognitive techniques for insomnia in 2018^22^ showed that PI has empirical support as an insomnia treatment. This was followed by the first comprehensive systematic review and meta-analysis of studies that explore the efficacy of PI for insomnia.^9^ In this review, 10 trials examining PI as a sole intervention were identified. Overall, the beneficial effects for PI were larger when compared with passive rather than active comparators, with the largest being obtained for difficulty falling asleep and the smallest for sleep onset latency. Relative to passive comparators, PI significantly improved sleep onset latency, difficulty falling asleep, number of awakenings, restedness and increased total sleep time with a negligible effect size on sleep efficiency. Compared with active comparators, PI demonstrated moderate improvements in difficulty falling asleep, number of awakenings, and restedness, and negligible effect sizes on sleep onset latency and total sleep time.^9^ A reasonable conclusion from this literature would be to state that PI tentatively has similar efficacy as other cognitive and behavioural interventions for insomnia.

### Limitations of previous research on PI

Quantitative data from three studies in the meta-analysis showed that PI was largely superior to passive comparators on a proposed mechanism of change: sleep performance anxiety. This finding strengthens the notion that decreased performance anxiety is a mechanism through which PI might work. One large concern is that in the three studies’ statistical analyses, the proposed mechanisms-of-change variables are examined as dependent variables; as such, they do not indicate whether changes in psychological processes (eg, performance anxiety) are mechanisms of change in PI outcomes. Performing mediational analyses would further advance the field by clarifying whether insomnia improvements are a result of changes in theorised variables. Also, as the theoretical development regarding insomnia has developed further since these studies were conducted, there are other processes that might explain how PI works, such as acceptance and metacognitions.^23^ With these limitations considered, new studies are needed that examine mechanisms-of-change variables with an appropriate statistical approach and measure all proposed mechanisms of change.

Several other important limitations and uncertainties with the empirical literature on PI were also identified.^9^ Among them were an almost exclusive focus on participants meeting criteria for sleep-onset insomnia or primary insomnia and thereby limiting knowledge of other insomnia groups, such as those with sleep maintenance issues or insomnia comorbid with other psychiatrics conditions which is common in the population.^24^ Also, previous studies largely lack information on how PI was administered (eg, training of therapists) and other important treatment-relevant domains pertaining to patients’ experiences (acceptability, adherence, credibility and expectancy ratings, and perceived usefulness of PI). Additionally, too few trials assessed the long-term efficacy of PI. All these limitations hamper the possibility of drawing strong conclusions about the efficacy of PI.

### Purpose and significance

The most recent meta-analysis supports PI as an effective intervention for insomnia disorder. However, some important aspects of PI have been insufficiently examined, including a modern high-quality assessment of its efficacy, its mechanisms of change and whether patient characteristics predict the treatment effects. The present uncontrolled pilot trial assesses the preliminary efficacy of this treatment on the Insomnia Severity Index (ISI) and secondary outcomes such as quality of life, depression and anxiety. This evaluation serves as groundwork for a possible larger scale randomised controlled trial. It is the first study to examine the effects of PI on insomnia severity using the ISI as well as to comprehensively evaluate secondary outcomes.

While Cognitive Behavioural Therapy for Insomnia (CBT-I) is the preferred first-line treatment for insomnia, there are accessibility issues which prevent many patients from receiving it.^1^ Additionally, research has shown challenges in retaining patients in CBT-I treatments.^25^ In this context, it should be pointed out that PI for insomnia was originally intended for patients who had not improved sufficiently after behavioural interventions for insomnia.^5^ At face value, PI offers a simple, scalable and possibly more acceptable treatment alternative, with a focus on eliminating the behaviours and beliefs that disrupt sleep without demanding greater changes in sleep habits such as sleep restriction. Additionally, there has been a call for increased research into the cognitive aspects of insomnia due to the scarcity of studies in this area.^22^ Consequently, this study could provide practitioners with new methods for the psychological management of insomnia.

This project aims to evaluate PI for insomnia by addressing four key areas: first, the preliminary efficacy of PI on sleep at post-treatment and 3-month follow-up; second, potential mediators of change to identify which processes are altered during PI that may influence treatment outcomes; third, patient characteristics that could moderate the response to PI; fourth, the acceptability and feasibility of PI as perceived by patients. Specifically, the pilot study will assess the immediate and sustained impact of PI on primary (Insomnia Severity Index; ISI) and secondary outcomes, including functional impairment, quality of life, depression, anxiety and sleep diary parameters. Additionally, it will investigate treatment-related aspects such as compliance and adverse events, explore the proposed mechanisms behind PI’s efficacy, determine the influence of patient characteristics on treatment outcomes and capture participants’ perceptions of PI through qualitative interviews.

## Methods and analysis

### Participants and procedures

This planned pilot trial without control condition aims to recruit 40 individuals with insomnia disorder through advertisements in newspapers, clinics and university-related settings across Sweden including campuses and social media. The sample size was determined based on recommendations for single-group pilot studies, which typically suggest 20–25 participants.^26^ Considering potential attrition of approximately 25% during the study period,^27^ and the uncertainty regarding the magnitude of effect sizes of PI for the primary outcome (ISI) and the mediators,^9^ we determined that 40 participants would be appropriate to achieve our aims of assessing the effects, acceptability and feasibility of the trial in preparation for a potential larger scale controlled trial. To be eligible for this trial, participants will undergo three screening phases that consist of first, a questionnaire on the web; second, a telephone interview with a structured assessment of inclusion and exclusion criteria using a standardised format, conducted by clinical psychologists or last year master students in psychology; and finally, the completion of a sleep diary (7 days). The criteria used for inclusion and exclusion in the three screening phases are based on expert recommendations for a standard research assessment of insomnia and the Diagnostic and Statistical Manual of Mental Disorders, Fifth Edition (table 1); noteworthy is that all forms of insomnia will be allowed in the study, including comorbid insomnia, which is more common than insomnia without other comorbid disorders.^1^

**Table 1:** Inclusion and exclusion criteria

Inclusion criteria	Exclusion criteria
Insomnia disorder (according to DSM-5 criteria) with or without psychiatric comorbidity	Severe depressive episode with suicidal intentions or actions
Age over 18Swedish resident and Swedish language proficient	Recent changes in psychopharmacotherapy (within the last 3 months) or use of ‘as-needed’ hypnotic medications during the trial
	Current or past diagnosis of psychotic or bipolar disorderCurrent substance use disorder

DSM-5Diagnostic and Statistical Manual of Mental Disorders, Fifth Edition

Following screening, the individuals offered to be part of the study will be presented with informed consent forms via the internet therapy platform and given the chance to ask questions verbally and in writing before signing. The participants who sign the informed consent form will be given baseline measures including checks for therapy credibility and expectancy and questions about concomitant treatments. Weekly outcome and process measures will follow in combination with treatment and at termination, checks for treatment adherence, treatment compliance and adverse events will take place. Throughout the 4-week treatment, weekly short (15 min or less) meetings with a therapist will take place via phone or videocalls. These meetings have the intention of offering individual support on the use of PI as well as attempting to solve any issues the participant might face with regard to implementing PI correctly.

### Paradoxical intention intervention

Each participant will undergo a 4-week paradoxical intention treatment. The decision to use a 4-week intervention period was based on a review of previous studies, where the typical duration for PI treatment was around four sessions.^9^ The intervention will be delivered via the internet through four weekly modules, with each module containing educational texts, instructions for minor exercises and homework assignments.

The first week’s module provides an overview of the treatment as well as information about insomnia, and exercises. It introduces the rationale behind the paradoxical intention technique and guides participants in how to implement the technique in the upcoming week. In the second module, participants will find information about common challenges encountered while applying the paradoxical intention technique. This module also offers support and ongoing encouragement to use the technique nightly. The third module continues with troubleshooting advice for any issues that may arise and further encourages participants to persist with the technique. The final, fourth module focuses on relapse prevention. Here, patients will receive information about maintaining their progress and strategies to respond to any potential setbacks. The 3-month follow-up will require participants to complete the set of measures outlined in table 2. A 3-month follow-up was selected to balance the need for assessment of longer-term effects while also minimising attrition which is critical given the smaller pilot sample. Few prior studies on PI have included follow-up assessments.^9^ The duration chosen was considered to be proportionate to the length of intervention, allowing for evaluation of the sustained effects without excessive participant drop-out.

**Table 2:** Overview of the study instruments and survey timepoints

Domain	Instrument	Inclusion screening	Baseline	W1	W2	W3	W4	FU 3 months
Insomnia outcome measures	ISI	x	x	x	x	x	x	x
Secondary outcome measures	Sleep diary	x	x		x		x	x
Secondary outcome measures	DASS-21		x		x		x	x
Secondary outcome measures	WSAS		x				x	x
Secondary outcome measures	BBQ		x				x	x
Proposed mechanisms	SAS		x		x		x	x
Proposed mechanisms	GSES		x		x		x	x
Proposed mechanisms	MCQ-I		x		x		x	x
Proposed mechanisms	SPAQ		x		x		x	x
Satisfaction with treatment	CSQ						x	
Expectations and credibility	CEQ			x				
Adverse effects	NEQ						x	
Eligibility screening, demographic information and informed consent	Constructed items	x						
Items on previous treatments, participants’ experience and engagement in treatment	Constructed items	x					x	

BBQ, Brunnsviken Brief Quality of life; CEQ, Credibility and Expectancy Questionnaire; CSQ, Client Satisfaction Questionnaire; DASS-21, Depression Anxiety Stress Scale-21; FUfollow-upGSES, Glasgow Sleep Effort Scale; ISI, Insomnia Severity Index; MCQ-I, Metacognitions Questionnaire-Insomnia; NEQNegative Effects QuestionnaireSAS, Sleep Anxiety Scale; Sleep diaryideographic sleep diarySPAQ, Sleep Problem Acceptance Questionnaire; WweekWSAS, Work and Social Adjustment Scale

### Qualitative interviews

Qualitative interviews will be conducted following the treatment, wherein 10–15 participants will be invited for interviews about the treatment. The exact number will be determined during the process, based on when information saturation has been achieved.^28^ These interviews, which can be carried out either in person or via videocalls, are not conducted by the treatment providers in order to mitigate the risk of social desirability bias from the participants. The interviews will be audio-recorded and transcribed for subsequent analysis using thematic analysis. The focus of these interviews will explore specific aspects such as participants’ sleep patterns during the treatment, their experiences with paradoxical intention, and various elements like text volume, therapist support and homework assignments. Questions will explore how these elements influenced their sleep quality and adherence to the treatment. Follow-up questions will probe deeper into their personal experiences, challenges faced and suggestions for improvement. To select interviewees, participants will be asked if they wish to partake in qualitative interviews and among those who consent, a selection will be made with the purpose of achieving a variety of expressed voices, for example, a variety of ages, genders and clinical outcomes represented.

### Measures

The timepoints of the different assessments used are summarised in table 2 following the Standard Protocol Items: Recommendations for Interventional Trials (SPIRIT) requirements. The full SPIRIT checklist with extended answers is provided in online supplemental material 1. All measures were either previously validated in Swedish or were translated from a validated language version to Swedish using back-translation.^29^

#### Primary outcomes

The ISI^30^ will be the primary outcome measure. The ISI is a 7-item scale that assesses the severity of insomnia symptoms. Each item is scored on a 5-point Likert scale, with higher scores indicating greater insomnia severity. The total score helps categorise the severity of insomnia (eg, no insomnia, subthreshold, moderate or severe).

#### Secondary outcomes

The following additional outcome areas will be measured: sleep diary parameters (ie, sleep onset latency, sleep regularity, sleep efficiency and sleep duration). Data from sleep diaries are recorded daily by the participants on the web platform, where averages are computed to assess sleep patterns. Depression and anxiety will be measured with the Depression Anxiety Stress Scale-21. Each item is rated on a 4-point scale, and scores are summed to yield a total score for each domain.^31^ Functional impairment is measured with the Work and Social Adjustment Scale (WSAS)^32^ which contains five items that evaluate functional impairment due to psychological or physical problems in work, social life and leisure activities. Each item is scored on an 8-point scale with higher scores indicating greater impairment. Quality of life will be measured with the Brunnsviken Brief Quality of life measure (BBQ).^33^ The BBQ contains 12 items that assess quality of life across various domains, such as learning, creativity and leisure. Items are rated on a 5-point scale with higher scores indicating better quality of life. To minimise participant fatigue, the WSAS and the BBQ will not be measured at mid-point of treatment (week 2).

#### Mechanisms and predictors

The following mechanisms of change will be measured. Sleep-related performance anxiety (Sleep Anxiety Scale; SAS).^12^ The SAS is an 8-item scale measuring anxiety specifically related to sleep. Items are rated on a 3-point scale with higher scores reflecting greater sleep-related anxiety. Sleep effort will be measured with the Glasgow Sleep Effort Scale,^34^ a 7-item scale assessing the extent of effort exerted to initiate or maintain sleep. Items are rated on a 3-point scale, with higher scores indicating more effort regarding sleep. Sleep Problem Acceptance Questionnaire^35^ is an 8-item scale that evaluates acceptance of sleep difficulties and willingness to engage in life despite them. Items are rated on a 7-point scale with higher scores indicate greater acceptance and less avoidance of sleep issues. Metacognitions Questionnaire-Insomnia Short Version,^36^ a 20-item scale that assesses metacognitions related to sleep and insomnia and its consequences. Items are rated on a 4-point scale; higher scores indicate more dysfunctional metacognitions. To investigate predictors for PI, the following areas will be assessed and screened at pre-treatment: sociodemographics (ie, age, gender, educational attainment, marital status and employment status), insomnia symptoms, use of other sleep treatments (eg, pharmacological), and psychiatric comorbidity and medical comorbidity (assessed using sections of the MINI clinical interview^37^ as well as self-reported prior diagnoses).

#### Additional measures

To analyse treatment acceptability and credibility, the Credibility and Expectations Questionnaire (CEQ),^38^ which is a 6-item measure that assesses participants’ expectations and perceived credibility of the treatment on a 9-point scale with higher scores suggesting greater belief in treatment efficacy and higher expectations to improve, will be measured at week 1 following the first module.

Additionally, the Client Satisfaction Questionnaire (CSQ)^39^ will be administered at post-treatment. The CSQ is an 8-item measure of satisfaction with treatment. Each item is rated on a 4-point scale where higher scores indicate greater satisfaction with the treatment offered. Finally, the Negative Effects Questionnaire (NEQ),^40^ a 20-item self-report measure assessing any negative effects experienced during treatment, including the client’s belief about whether these effects were treatment-related or due to external factors, and the extent of their negative impact will be administered once at post-treatment.

### Data analysis

All primary and secondary outcome data will be analysed using linear mixed models which are able to handle missing data and dependency well.^41^ The analyses will follow a stepwise approach, first examining changes from baseline to postintervention to assess the effects of the PI treatment. Then, changes from postintervention to follow-up will be analysed to determine whether the effects are maintained over time. The mixed-models approach will be used to estimate parameters, selecting the best-fitting model based on information criteria comparisons. An alpha level of 0.05 will be used as the criterion for statistical significance. Missing data will be treated as ‘missing at random’ using intent to treat analysis. As the planned study is a pilot, primary and secondary outcomes are only to be interpreted as preliminary results.

Regarding the process measures and proposed mechanisms, the SPSS macro MEMORE for repeated measures bootstrap analysis with multiple proposed mediators^42^ will be employed to investigate baseline to postintervention changes on the proposed mediators (sleep performance anxiety, sleep effort, acceptance and metacognitions) of significant pre-intervention to postintervention primary outcomes. Baseline and postintervention scores for a significant primary outcome measure (Y) and each proposed mediator (M) will be entered with (X) representing the interval of time between measurements. Bias-corrected and accelerated (BCa) bootstrap confidence intervals will be employed. Mediation is significant if the 95% BCa for the indirect effects does not include zero.^43^

The mediational analyses will also explore if different predictors, such as baseline insomnia severity, comorbidity and other patient characteristics, may affect this relationship as time-invariant covariates.

Descriptive statistics such as the percentage of participant attendance and dropout rates as well as the mean and distribution of CEQ and NEQ will be used to analyse the quantitative outcomes of acceptability, feasibility and credibility. Significance levels will be set at p<0.05 for all quantitative statistics tests and effect sizes (Cohen’s d) will be calculated.

The qualitative data from post-treatment interviews will be analysed using thematic analysis.^44^ Initial codes will be generated and grouped into larger themes depending on the emerging data. The number of qualitative interviews will be about 10 depending on when information saturation is reached.

### Patient and public involvement

This study was designed without direct patient or public input. However, the authors drew on their clinical experience using PI in CBT-I group therapies, alongside previous literature and theoretical frameworks, to shape the research questions and select outcome measures. The design addresses research gaps and common concerns among individuals with insomnia disorder. By using a mixed-methods approach, we aim to gain a comprehensive understanding of the patient’s experience with PI. The qualitative and quantitative results of this pilot trial will guide protocol and practice adjustments for future studies. Participants will engage in weekly therapist meetings to monitor progress. Results will be published in an open-access journal, although specific dissemination plans for patients or the public are not included.

## Ethics and dissemination

Ethical approval by the Swedish Ethical Review Authority was attained before starting data collection (Dnr: 2023-06594-01). All participants will sign informed consent forms prior to enrolment with opportunities to ask questions regarding their participation to the investigators. Any substantive changes to the protocol or the conduct of the study, which may impact participant safety, the integrity of the study or the potential benefits to participants, will be managed through a formal amendment process to the Swedish Ethical Review Authority as well as by updating the trial registration protocol in Clinicaltrials.gov (NCT06259682). Results will be disseminated through peer-reviewed publications. The findings will inform adaptations to the treatment protocol and serve as groundwork for a possible larger scale randomised controlled trial.

### Study timeline

The project is set to begin with the recruitment of participants during the autumn and winter of 2024. The recruitment process will continue throughout this period, allowing for the screening of participants as they register their interest. By the spring and summer of 2025, the participants who have been included will be assigned to begin their treatment. The goal is to ensure that all participants have been included and treated within the project’s framework by June 2025.

Following this, the autumn and winter of 2025 will focus on follow-up assessments. It is anticipated that all participants will complete their final follow-up, which is scheduled to occur 3 months after the end of treatment, within this period. The project will then move into the final phase during the spring and summer of 2026, where data analysis and manuscript writing will take place, culminating in the submission of findings to peer-reviewed journals.

## Discussion

This prospective pilot study also has several strengths. First among these is the mixed-methods approach, which allows for a deeper understanding of the treatment’s feasibility and acceptability, the qualitative interviews are thought in a later stage to be informative of subsequent alterations in the delivery and rationale of the technique which echoes a more recent interest in participatory research and adaptive designs.^45^ Second, the focus on mechanisms and predictors allows us greater precision in formulating why and under which circumstances PI might work. The fact that PI, in essence, only contains a single treatment component also enhances the possibility of claiming specificity of the mechanisms, although ideally, there would be a control condition with another single technique intervention. Third, the wider inclusion of patients with both singular and comorbid insomnia means that results are generalisable to a more representative sample since comorbidity seems to be the norm in most investigated populations.^1^

One of the primary limitations of this study is the uncontrolled design, which does not allow us to assess for the non-specific factors of treatment, which reasonably affects the improvement rates among participants. On the other hand, given that there have been no studies on PI during the last decade and more, conducting an uncontrolled pilot is a cost-effective and safe way of assessing this technique’s preliminary efficacy as well as the acceptability and feasibility of the approach. In the same vein, while the small sample size might be seen as a limitation, as a larger sample size would have allowed for greater power in analysing relationships between different process-variables, it would also have increased the number of participants who would be subjected to a treatment of unclear efficacy. Another limitation is that, although the inclusion criteria require that participants maintain stable and daily doses of their prescribed sleeping medications, the possibility cannot be ruled out that some participants may alter their doses without notifying the investigators. Additionally, the increased attention to their sleep patterns during the trial might lead to enhanced adherence to their psychopharmacological treatment. Such changes could influence sleep outcomes, potentially biasing the results. To mitigate this risk, the investigators will regularly remind participants to report any changes in their medication use. Finally, the sole use of self-reports as outcome measure could be seen as a limitation, given that many patients with insomnia tend to have substantial misperceptions regarding their own sleep efficiency and sleep onset.^46^ Indeed, as paradoxical intention explicitly aims for patients to remain awake, an even greater discrepancy between actual and perceived sleep onset latency might have occurred in some participants. The use of actigraphy or other objective measures of sleep onset might have allowed us to correct for possible such biases although it should be noted that some technological tools (eg, actigraphy) for measuring sleep onset can produce false results in sleep states, especially given the PI instructions of lying still in bed.^47^

If this study finds that PI leads to improvements in insomnia severity, it may warrant a randomised controlled trial against an active comparator. The utility of PI could be significant as it is a relatively easily administered technique that can supplement or be an alternative to established CBT-I treatments.^23^ Not all patients benefit from CBT-I,^48^ and, as such, there is a need for further psychological treatment alternatives that are scalable. Additionally, if further understanding of mechanisms of change are found through this study, then more specificity will have been added to the insomnia literature allowing researchers to experiment with tailoring interventions to maximise the effect on suggested mechanisms. This could be tried in a controlled trial vs another treatment or in a dismantling design where PI is added or removed from a larger protocol. In short, the establishment of mechanisms of change allows for greater precision in insomnia interventions.

## supplementary material

10.1136/bmjopen-2024-086676 online supplemental file 1
