# Supplementary material for: Paradoxical intention as a treatment for insomnia disorder: study protocol for a mixed-methods pilot trial
Source: BMJ Open. 2024 Oct 10;14(10):e086676. doi: 10.1136/bmjopen-2024-086676 (PMC11474766; doi:10.1136/bmjopen-2024-086676)
Supplement: online supplemental file 1 [file bmjopen-14-10-s001.pdf]

SPIRIT

STANDARD PROTOCOL ITEMS: RECOMMENDATIONS FOR INTERVENTIONAL TRIALS

SPIRIT 2013 Checklist: Recommended items to address in a clinical trial protocol and related documents*

Section/item	Item No	Description	Addressed on page number
**Administrative information**			
Title	1	Descriptive title identifying the study design, population, interventions, and, if applicable, trial acronym	______1_________
Trial registration	2a	Trial identifier and registry name. If not yet registered, name of intended registry	______2_________
	2b	All items from the World Health Organization Trial Registration Data Set	Supplementary material 1
Protocol version	3	Date and version identifier	Supplementary material 1
Funding	4	Sources and types of financial, material, and other support	______1_________
Roles and responsibilities	5a	Names, affiliations, and roles of protocol contributors	______1_________
	5b	Name and contact information for the trial sponsor	Supplementary material 1
	5c	Role of study sponsor and funders, if any, in study design; collection, management, analysis, and interpretation of data; writing of the report; and the decision to submit the report for publication, including whether they will have ultimate authority over any of these activities	Supplementary material 1

	5d	Composition, roles, and responsibilities of the coordinating centre, steering committee, endpoint adjudication committee, data management team, and other individuals or groups overseeing the trial, if applicable (see Item 21a for data monitoring committee)	__Not Applicable
**Introduction**			
Background and rationale	6a	Description of research question and justification for undertaking the trial, including summary of relevant studies (published and unpublished) examining benefits and harms for each intervention	______3-7______
	6b	Explanation for choice of comparators	______7-8_____
Objectives	7	Specific objectives or hypotheses	______7-8______
Trial design	8	Description of trial design including type of trial (eg, parallel group, crossover, factorial, single group), allocation ratio, and framework (eg, superiority, equivalence, noninferiority, exploratory)	______7-8___
**Methods: Participants, interventions, and outcomes**			
Study setting	9	Description of study settings (eg, community clinic, academic hospital) and list of countries where data will be collected. Reference to where list of study sites can be obtained	______8_______
Eligibility criteria	10	Inclusion and exclusion criteria for participants. If applicable, eligibility criteria for study centres and individuals who will perform the interventions (eg, surgeons, psychotherapists)	_____8 (table 1)
Interventions	11a	Interventions for each group with sufficient detail to allow replication, including how and when they will be administered	______9_______
	11b	Criteria for discontinuing or modifying allocated interventions for a given trial participant (eg, drug dose change in response to harms, participant request, or improving/worsening disease)	__Not Applicable
	11c	Strategies to improve adherence to intervention protocols, and any procedures for monitoring adherence (eg, drug tablet return, laboratory tests)	__Not Applicable
	11d	Relevant concomitant care and interventions that are permitted or prohibited during the trial	_____8 (table 1)

Outcomes	12	Primary, secondary, and other outcomes, including the specific measurement variable (eg, systolic blood pressure), analysis metric (eg, change from baseline, final value, time to event), method of aggregation (eg, median, proportion), and time point for each outcome. Explanation of the clinical relevance of chosen efficacy and harm outcomes is strongly recommended	_8-11 (incl table 2)
Participant timeline	13	Time schedule of enrolment, interventions (including any run-ins and washouts), assessments, and visits for participants. A schematic diagram is highly recommended (see Figure)	_____Table 2__
Sample size	14	Estimated number of participants needed to achieve study objectives and how it was determined, including clinical and statistical assumptions supporting any sample size calculations	_____8___
Recruitment	15	Strategies for achieving adequate participant enrolment to reach target sample size	______8_______
**Methods: Assignment of interventions (for controlled trials)**			
Allocation:			
Sequence generation	16a	Method of generating the allocation sequence (eg, computer-generated random numbers), and list of any factors for stratification. To reduce predictability of a random sequence, details of any planned restriction (eg, blocking) should be provided in a separate document that is unavailable to those who enrol participants or assign interventions	__Not applicable
Allocation concealment mechanism	16b	Mechanism of implementing the allocation sequence (eg, central telephone; sequentially numbered, opaque, sealed envelopes), describing any steps to conceal the sequence until interventions are assigned	__Not applicable
Implementation	16c	Who will generate the allocation sequence, who will enrol participants, and who will assign participants to interventions	__Not applicable
Blinding (masking)	17a	Who will be blinded after assignment to interventions (eg, trial participants, care providers, outcome assessors, data analysts), and how	__Not applicable
	17b	If blinded, circumstances under which unblinding is permissible, and procedure for revealing a participant's allocated intervention during the trial	__Not applicable
**Methods: Data collection, management, and analysis**			

Data collection methods	18a	Plans for assessment and collection of outcome, baseline, and other trial data, including any related processes to promote data quality (eg, duplicate measurements, training of assessors) and a description of study instruments (eg, questionnaires, laboratory tests) along with their reliability and validity, if known. Reference to where data collection forms can be found, if not in the protocol	___9-12_
	18b	Plans to promote participant retention and complete follow-up, including list of any outcome data to be collected for participants who discontinue or deviate from intervention protocols	_____8-9____
Data management	19	Plans for data entry, coding, security, and storage, including any related processes to promote data quality (eg, double data entry; range checks for data values). Reference to where details of data management procedures can be found, if not in the protocol	Supplementary material 1
Statistical methods	20a	Statistical methods for analysing primary and secondary outcomes. Reference to where other details of the statistical analysis plan can be found, if not in the protocol	____12-13
	20b	Methods for any additional analyses (eg, subgroup and adjusted analyses)	____12-13
	20c	Definition of analysis population relating to protocol non-adherence (eg, as randomised analysis), and any statistical methods to handle missing data (eg, multiple imputation)	____12-13

Methods: Monitoring

Data monitoring	21a	Composition of data monitoring committee (DMC); summary of its role and reporting structure; statement of whether it is independent from the sponsor and competing interests; and reference to where further details about its charter can be found, if not in the protocol. Alternatively, an explanation of why a DMC is not needed	__Not Applicable
	21b	Description of any interim analyses and stopping guidelines, including who will have access to these interim results and make the final decision to terminate the trial	__Not Applicable
Harms	22	Plans for collecting, assessing, reporting, and managing solicited and spontaneously reported adverse events and other unintended effects of trial interventions or trial conduct	Supplementary material 1
Auditing	23	Frequency and procedures for auditing trial conduct, if any, and whether the process will be independent from investigators and the sponsor	__Not Applicable

Ethics and dissemination

Research ethics approval	24	Plans for seeking research ethics committee/institutional review board (REC/IRB) approval	_____13______
Protocol amendments	25	Plans for communicating important protocol modifications (eg, changes to eligibility criteria, outcomes, analyses) to relevant parties (eg, investigators, REC/IRBs, trial participants, trial registries, journals, regulators)	_____13______
Consent or assent	26a	Who will obtain informed consent or assent from potential trial participants or authorised surrogates, and how (see Item 32)	______8-9____
	26b	Additional consent provisions for collection and use of participant data and biological specimens in ancillary studies, if applicable	__Not Applicable
Confidentiality	27	How personal information about potential and enrolled participants will be collected, shared, and maintained in order to protect confidentiality before, during, and after the trial	Supplementary material 1
Declaration of interests	28	Financial and other competing interests for principal investigators for the overall trial and each study site	_____1________
Access to data	29	Statement of who will have access to the final trial dataset, and disclosure of contractual agreements that limit such access for investigators	_____17_______
Ancillary and post-trial care	30	Provisions, if any, for ancillary and post-trial care, and for compensation to those who suffer harm from trial participation	__Not Applicable
Dissemination policy	31a	Plans for investigators and sponsor to communicate trial results to participants, healthcare professionals, the public, and other relevant groups (eg, via publication, reporting in results databases, or other data sharing arrangements), including any publication restrictions	Supplementary material 1
	31b	Authorship eligibility guidelines and any intended use of professional writers	Supplementary material 1
	31c	Plans, if any, for granting public access to the full protocol, participant-level dataset, and statistical code	Title page.
**Appendices**			
Informed consent materials	32	Model consent form and other related documentation given to participants and authorised surrogates	Supplementary material 1

Biological specimens	33	Plans for collection, laboratory evaluation, and storage of biological specimens for genetic or molecular analysis in the current trial and for future use in ancillary studies, if applicable	__Not Applicable

*It is strongly recommended that this checklist be read in conjunction with the SPIRIT 2013 Explanation & Elaboration for important clarification on the items. Amendments to the protocol should be tracked and dated. The SPIRIT checklist is copyrighted by the SPIRIT Group under the Creative Commons "Attribution-NonCommercial-NoDerivs 3.0 Unported" license.

List 1. Below are elaborations and answers to the SPIRIT 2013 Checklist items indicated as addressed in "Supplementary material 1"

Item 2b: All items from the World Health Organization Trial Registration Data Set
Trial registration—data set

Data category	Information
Primary registry and trial identifying number	ClinicalTrials.gov ID: NCT06259682
Date of registration in primary registry	Last Update: 03/08/2024
Secondary identifying numbers	Unique Protocol ID: 2023-06594-01
Source(s) of monetary or material support	Rut och Arvid Wolfs Minnesstiftelse<br>Svensk Förening för Sömnförskning 2022
Primary sponsor	Örebro University Sweden
Secondary sponsor(s)	Karolinska Institutet
Contact for public queries	Annika Norell, Telephone: +4619302259, Email: Annika.Norell@oru.se
Contact for scientific queries	Annika Norell, Telephone: +4619302259, Email: Annika.Norell@oru.se
Public title	A Mixed-method Pilot Investigation of Paradoxical Intention for Insomnia
Scientific title	A Mixed-method Pilot Investigation of Paradoxical Intention for Insomnia: Assessing Acceptability, Feasibility and Preliminary Effectiveness
Countries of recruitment	Sweden
Health condition(s) or problem(s) studied	Insomnia
Intervention(s)	Behavioral: Paradoxical Intention
Inclusion criteria	Diagnosis of insomnia including co-morbid insomnia, fulfilling DSM-5 criteria, age above 18, Swedish resident and language proficient
Exclusion criteria:	Bipolar or psychotic disorders, suicidal intentions or actions, current substance abuse, recent changes in psychopharmacotherapy within the last three months
Study type	Interventional
Allocation	N/A (Single Group Assignment)
Primary purpose:	Treatment
Date of first enrolment	Study Start: April 2024 [Anticipated]
Target sample size	Enrollment: 40 [Anticipated]
Recruitment status	Overall Status: Not yet recruiting
Primary outcome(s)	Insomnia Severity Index (ISI) Sleep diary

Data category	Information
Key secondary outcomes	Work and Social Adjustment Scale (WSAS) Depression Anxiety Stress Scales (DASS) Brunnsviken Brief Quality of Life Scale (BBQ) Sleep Anxiety Scale (SAS) Glasgow Sleep Effort Scale (GSES) Metacognitions Questionnaire - Insomnia (MCQ-I) Sleep Problem Acceptance Questionnaire (SPAQ) Client Satisfaction Questionnaire (CSQ) Credibility/Expectancy Questionnaire (CEQ) Negative effects Questionnaire (NEQ)

Item 3: Date and version identifier

Protocol version

- Issue date: 20-03-2024.
- Protocol amendment number: 00
- Authors: *OS, MJF, CH, AN*

Item 5b: Name and contact information for the trial sponsor.

Roles and responsibilities—sponsor contact information

Trial Sponsor: Örebro University, Sweden

Sponsor's Reference: ORU 4.2-07945/2022.

Contact name: Annika Norell, Telephone: +4619302259, Email: Annika.Norell@oru.se

Address: Fakultetsgatan 1, 70281 Örebro. Sweden

Telephone: +4619302259

Email: Annika.Norell@oru.se

Item 5c: Role of study sponsor and funders, if any, in study design; collection, management, analysis, and interpretation of data; writing of the report; and the decision to submit the report for publication, including whether they will have ultimate authority over any of these activities.

Roles and responsibilities—sponsor and funder

Statement from authors OS, MJF, CH and AN: The funding sources had no role in the design of this study and will not have any role during its execution, analyses, interpretation of the data, or decision to submit results.

Item 19: Plans for data entry, coding, security, and storage, including any related processes to promote data quality (e.g., double data entry; range checks for data values). Reference to where details of data management procedures can be found, if not in the protocol.

Data management

For our study, all data will be collected and managed through the secure Internet therapy platform, Iterapi. This platform is designed to ensure the security and protection of participant data, requiring secure multifactorial passwords for participant access. This access control mechanism is critical for protecting the confidentiality of treatment modules and participant data within the platform.

Data Entry and Management:

Participants will input their data directly into Iterapi during their engagement with the psychological intervention modules. The platform is equipped with data validation rules to ensure the integrity of the data collected. This includes validation for data values at the point of entry.

Data Security and Protection:

To safeguard participant privacy and data confidentiality, the platform employs robust security measures. All data collected through Iterapi are pseudonymized before analysis, with identifiers replaced by unique codes. The linkage between these codes and actual participant identities is encrypted and stored separately on a password-protected USB drive, ensuring that data re-identification is strictly controlled and limited to authorized study personnel only.

Data Storage and Backup:

The pseudonymized data will be extracted from Iterapi for analysis, with backups stored securely and encrypted to prevent unauthorized access. Regular backups of the data will be conducted to ensure data integrity and availability, with all backups encrypted and stored in secure, access-controlled environments.

Quality Assurance:

Our data management plan includes procedures to promote data quality, such as periodic checks for data completeness and accuracy. Any discrepancies identified during these checks will be investigated promptly, with corrections made as necessary following a documented process to ensure data integrity.

This tailored approach to data management for our study, utilizing the Iterapi platform, ensures that we maintain the highest standards of data quality, security, and participant privacy.

Item 22: Plans for collecting, assessing, reporting, and managing solicited and spontaneously reported adverse events and other unintended effects of trial interventions or trial conduct

Harms

In our study, adverse events are primarily focused on any negative psychological effects or experiences that participants may encounter because of the intervention, with a particular emphasis on negative experiences reported through the Negative Effects Questionnaire at the end of treatment. Additionally, weekly short conversations with each participant, conducted by licensed healthcare personnel or those under their supervision, serve as a supplementary method for identifying and assessing any adverse events or worsening of the participant's condition. These conversations, along with weekly self-report ratings of symptoms, allow for the early detection of any negative impacts of the intervention, ensuring prompt action can be taken to address these issues.

Definition and Assessment of Adverse Events: An adverse event in our study is defined as any negative psychological effect or experience reported by the participant that is associated with the intervention or the trial conduct. This includes, but is not limited to, increased symptoms, emotional distress, or any other psychological discomfort. Licensed healthcare personnel will evaluate the severity and causality of reported adverse events, taking into account the temporal relationship to the intervention and the participant's overall psychological condition.

Collection of Adverse Events: Adverse events will be collected through several methods:

- **Negative Effects Questionnaire:** Administered at the end of treatment to systematically capture any negative experiences related to the intervention.
- **Weekly Conversations:** Licensed healthcare personnel will conduct brief check-ins with participants to discuss their progress and any negative experiences.
- **Weekly Symptom Ratings:** Participants will provide self-reported ratings of their symptoms, which will be monitored for indications of worsening conditions.

Management of Adverse Events: Upon identification of an adverse event, the responsible healthcare personnel will take appropriate action based on the severity and nature of the event. This may include:

- Adjustments to the intervention,
- Provision of additional support,
- Referral to other healthcare services if a participant is deemed to be at risk or if the intervention is considered to be causing harm.

Analysis and Dissemination: Negative effects data will be reported to identify patterns or trends in the occurrence of negative effects associated with the intervention. The analysis will contribute to understanding the intervention's safety profile and will be included in the final study report and any publications, ensuring transparency and informing future clinical practice.

This comprehensive approach to managing and reporting adverse events is designed to safeguard participant well-being throughout the study, ensuring that any negative effects are promptly addressed and contributing valuable insights into the intervention's safety profile.

Item 27: How personal information about potential and enrolled participants will be collected, shared, and maintained in order to protect confidentiality before, during, and after the trial.

Confidentiality

Data Collection and Pseudonymization:

- All participant data, including self-reported measures and qualitative data, will be collected through secure, encrypted online platforms or through secure communications with participants.
- Upon collection, all personal identifying information (PII) will be pseudonymized. This process involves replacing PII with a unique code and anonymizing the data while allowing for data analysis.
- The key linking participants' PII to their unique codes will be stored securely on a password-protected USB drive, accessible only to authorized study personnel.

Data Storage and Security:

- All data, both pseudonymized and the separate key file, will be stored on encrypted, password-protected platforms. Physical copies of any data or consent forms will be kept in locked cabinets, in areas with restricted access.
- Digital data will be stored on secure university servers with limited access with protected firewalls, encryption, and password protocols.
- Access to sensitive or personal data will be controlled, limited to those staff members who require it for the purpose of the study, and monitored through a log of access instances.

Confidentiality during Analysis and Reporting:

- During data analysis and reporting, all information will be presented in a manner that ensures participants cannot be individually identified. Any potentially identifying details will be omitted or altered without compromising the integrity of the data.
- In publications or presentations, data will be aggregated or anonymized to further protect participant confidentiality.

Transmission of Data:

- Any necessary transmission of data, whether for analysis by team members or reporting to regulatory bodies, will be conducted through secure, encrypted channels to prevent unauthorized access or interception.

Consent and Information Sharing:

- Participants will be informed about the data collection, pseudonymization process, and measures taken to ensure their confidentiality through the informed consent process.

- Written permission will be obtained from participants before any of their personal information is released outside of the study team, except as required by law or for monitoring purposes by authorized entities such as ethics review boards or regulatory agencies.

Post-Trial Data Maintenance:

- Following the conclusion of the trial, all data will be maintained in accordance with university regulatory requirements for a period specified by local regulations, after which it will be securely destroyed or archived, depending on the data type and ethical guidelines..

Item 31a: Plans for investigators and sponsor to communicate trial results to participants, healthcare professionals, the public, and other relevant groups (e.g., via publication, reporting in results databases, or other data sharing arrangements), including any publication restrictions

Dissemination policy—trial results

Publication in Open Access Journals:

- We aim to publish the results of our study in peer-reviewed, open access journals to ensure that our findings are readily accessible to both the scientific community and the public. This approach facilitates the widespread dissemination of knowledge and contributes to evidence-based practice.

Possible presentations:

- Depending on the circumstances of the project, we may also publish a preprint of our findings.
- We plan to present the results of our study at relevant conferences, both as posters and lectures.

No Publication Restrictions:

- Our protocol states no restrictions on the publication or presentation of trial results. We believe that all findings, whether positive, negative, or null, should be made public to contribute to the body of knowledge in psychological interventions and inform future research and practice.

Ethical Considerations and Timelines:

- In line with ethical guidelines, we will ensure that any dissemination of results, including publications and presentations, respects the confidentiality and privacy of our participants.
- We anticipate taking a few months post-data collection to analyze the results comprehensively. Following analysis, we will promptly proceed with the submission of our findings to an appropriate journal and prepare summaries for dissemination among participants and other stakeholders.

Item 32: Model consent form and other related documentation given to participants and authorised surrogates

Informed consent materials

Information om forskningsprojektet <u>Paradoxal intention vid sömnlöshet</u>

Bakgrund och syfte

Insomni är en vanlig form av sömnbesvär som kännetecknas av att man har svårt att somna på kvällen, vaknar upp på natten och har problem att somna om eller vaknar upp tidigare än vad som är önskat på morgonen med problem att somna om. Kännetecknande för insomni är också att man upplever besvär på dagen, såsom trötthet, sömnighet, nedstämdhet och funktionsnedsättning i sin vardag.

Det huvudsakliga syftet med forskningsprojektet är att studera effekten av paradoxal intention. Paradoxal intention har använts som en psykoterapiteknik sedan 60-talet. Behandlingen handlar om att hitta ett alternativt sätt att förhålla sig till sina tankar och sin egen sömn. Paradoxal intention är en metod som ibland ingår i behandlingsformen kognitiv beteendeterapi (KBT). I det aktuella projektet förmedlas paradoxal intention via internet. KBT förmedlad via internet är något som under tio år har visat sig ha jämförbara effekter med traditionell KBT där terapeut och klient träffas. Alla deltagare som uppfyller studiens samtliga kriterier kommer att erbjudas paradoxal intention via internet.

Forskningshuvudman för projektet är Örebro Universitet. Med forskningshuvudman menas den organisation som är ansvarig för projektet. Projektet är godkänd av Etikprövningsmyndigheten, diarienummer för prövningen hos Etikprövningsmyndigheten är 2023-06594-01. Ditt deltagande i projektet är kostnadsfritt.

Förfrågan om deltagande

Vi söker dig som...

- Är svensk medborgare, talar och läser svenska, samt är minst 18 år
- Uppfyller projektets kriterier för insomni
- Har tillgång till dator, mobiltelefon, e-postadress och internet

Du ska inte...

- Genomgå eller tidigare ha genomgått paradoxal intention för sömnbesvär
- Lida av en djup depression

Du kan delta även om du...

- Genomgår läkemedelsbehandling så länge som dosen har varit stabil sedan tre månader

Hur går forskningsprojektet till?

Vi kommer att stämma av att du uppfyller studiens samtliga kriterier i tre steg: (1) du svarar på frågor under 20 minuter på vår tvåstegsskyddade websida, (2) någon i forskargruppen intervjuar dig via telefon eller digitalt rum under 30-60 minuter och (3) du svarar på en sömndagbok under en vecka, vilket tar cirka 5 minuter per dag.

Behandlingen ges via internet, och innebär att du en gång i veckan i fyra veckor får läsa en text och utföra hemuppgifter. Under hela behandlingen har varje deltagare veckovis avstämning med en psykolog, psykologstudent eller legitimerad sjuksköterska som hjälp. Denna kontakt sker via telefon, videomöte eller i vissa fall fysiskt på Örebro Universitet beroende på önskemål och behandlarens tillgänglighet. Vi kan tyvärr inte garantera att kontaktformen för din behandling kommer matcha dina önskemål. Behandlingen innefattar en hel del arbete på egen hand, och att det krävs ett aktivt engagemang under hela behandlingsperioden.

Tackar du ja till att delta i projektet kommer du få fylla i enkäter före och efter behandlingen. I enkäterna kommer du få svara på frågor som berör din hälsa och den beräknade tiden för att fylla i enkäter är 25 minuter före behandlingen, 15 minuter efter behandlingen, 15 min per vecka under behandlingen samt delta i en intervju à 30 minuter direkt efter avslutad behandling – totalt ca 85 minuter under hela studien över ca 2 månader.

Vilka är riskerna?
Förändring av sömnvanor och sömnmönster kan inledningsvis medföra en ökad trötthet. Inga allvarliga biverkningar av behandlingen är kända. Att besvara enkäter och att bli intervjuad kan uppfattas som besvärande och tidskrävande. Du avgör själv vilka frågor du vill svara på. Du har möjlighet att avbryta ditt deltagande i forskningsprojektet närhelst du önskar utan att uppge orsak och utan att det påverkar din övriga vård. Vi bedömer att fördelarna med behandling överväger eventuella risker och obehag. Upplever du problem som rör projektet ber vi dig ta kontakt med ansvarig forskare.

Finns det några fördelar?
För dig som har återkommande svårigheter med insomni kan den behandling som du får leda till en förbättring. Det skulle då kunna hjälpa dig hantera dina besvär framöver.

Hantering av data och sekretess
Allt material hanteras i avidentifierad form och i ett slutet kontakthanteringssystem genom tvåstegsverifiering, med lösenord och kod. Även inne i behandlingsprogrammet skyddas din identitet med tvåstegsverifiering. De data som du bidrar med i projektet lagras i en skyddad server på Örebro Universitet, där personuppgifter (t.ex. namn) inte framgår. Den kodnyckel som knyter samman dina data och personuppgifter förvaras på en annan skyddad server på Örebro Universitet.

Vi följer EU:s dataskyddsförordning. All information och alla uppgifter som samlas in i detta forskningsprojekt kommer att hanteras under sekretess i behandlingsfasen. Efter att datainsamlingen är klar kommer all information anonymiseras och kodas. Vidare kommer endast forskargruppen, genom inloggning med tvåstegsverifiering, ha behörighet till den insamlade datan. (d.v.s. inga obehöriga kommer kunna ta del av informationen eller identifiera enskilda individer). Du har rätt att en gång per år, gratis, ta del av de personuppgifter som behandlas om dig. Du kan också begära att uppgifter om dig raderas samt att behandlingen av dina personuppgifter begränsas. Rätten till radering och till begränsning av behandling av personuppgifter gäller dock inte när uppgifterna är nödvändiga för den aktuella forskningen. Om du är intresserad av ditt individuella resultat eller dina uppgifter ber vi dig att kontakta projektets kontaktperson Annika Norell (se info nedan). Dataskyddsombud nås på dataskyddsombud@oru.se. Om du är missnöjd med hur dina personuppgifter behandlas har du rätt att ge in klagomål till Integritetsskyddsmyndigheten, som är tillsynsmyndighet.

Rättslig grund för vår behandling av dina personuppgifter är allmänt intresse då forskningen har som syfte att utveckla vården vilket gagnar allmänheten.

Framkommer det att det står något felaktigt om dig skall den uppgiften rättas. Efter 15 år kommer kodnyckeln att förstöras, därefter är det inte längre möjligt att lämna ut någon information.

Material som samlas in genom frågeformulär och sömndagböcker sker i avidentifierad form, men blir tillfälligt synlig för den terapeut som kommer följa dig varje vecka under

behandlingen. Detta görs för att terapeuten ska kunna se hur det går för dig och för att möjliggöra att behandlingen kan skräddarsys för att passa dig bäst.

Vi kommer att sammanfatta resultatet på grupp och individuellnivå men det kommer inte vara möjligt att identifiera enskilda individer från deras deltagande. Det betyder att inga uppgifter som redovisas kommer att gå att spåra till dig personligen.
Resultatet kommer att presenteras i internationella vetenskapliga tidskrifter och eventuellt i form av examensuppsatser vid Örebro Universitet.

Försäkring och ersättning
Ingen ersättning betalas ut för ditt deltagande, men behandlingen erbjuds utan kostnad, och det ingår inte heller någon särskild försäkring för att du deltar.

Frivillighet
Ditt deltagande i forskningsprojektet är helt frivilligt och du kan när som helst välja att avbryta din medverkan. Om du väljer att avbryta ditt deltagande behöver du inte uppge anledningen till ditt beslut, och det kommer inte att påverka dina möjligheter att få tillgång till någon annan vård. Väljer du att avbryta ditt deltagande så uppskattas det om du meddelar detta till någon av de ansvariga.

Ansvarig forskare:
Docent & Leg.Psykolog Annika Norell
Örebro Universitet, Institutionen för
beteende-, social- och rättsvetenskap
Örebro, Sweden
Annika.Norell@oru.se

SAMTYCKE TILL ATT DELTA I STUDIEN ”Paradoxal intention vid sömnlöshet”

Jag har fått muntlig och/eller skriftlig information om studien och har haft möjlighet att ställa frågor. Jag får behålla den skriftliga informationen.

- Jag samtycker till att delta i projektet ”Paradoxal intention vid sömnlöshet”

Plats och datum	Underskrift
	Namnförtydligande
