# Supplementary material for: Paradoxical intention as a treatment for insomnia disorder: study protocol for a mixed-methods pilot trial
Source: BMJ Open. 2024 Oct 10;14(10):e086676. doi: 10.1136/bmjopen-2024-086676 (PMC11474766; doi:10.1136/bmjopen-2024-086676)
Supplement: Supplementary file 2 [file bmjopen-2024-086676.reviewer_comments.pdf]

PEER REVIEW HISTORY

BMJ Open publishes all reviews undertaken for accepted manuscripts. Reviewers are asked to complete a checklist review form and are provided with free text boxes to elaborate on their assessment. These free text comments are reproduced below.

ARTICLE DETAILS

Title (Provisional)

Paradoxical intention as a treatment for insomnia disorder: study protocol for a mixed-methods pilot trial

Authors

Salim, Osame; Jansson-Fröjmark, Markus; Sandlund, Christina; Norell, Annika

---

VERSION 1 - REVIEW

**Reviewer**	**1**
**Name**	**Bao, Yanping**
**Affiliation**	**Peking University**
**Date**	**13-Jul-2024**
**COI**	**No**

Thank you for giving me this opportunity to review this manuscript. There are some concerns before publication:

1. Author mentioned”Process and outcome measures will be assessed weekly across a 4-week intervention period and at a 3-month follow-up”.Therefore, the time setting of the intervention period and follow-up period and the reasons for such setting should be elaborated in the methods section of this paper.

2. The purpose of this project is to evaluate the initial efficacy of PIPI for short - and long-term sleep. Please describe the basis and criteria of short - and long-term sleep.

3. For the scale or questionnaire mentioned in the paper, there should be an introduction to the items, scoring principles and other information.

4. In secondary outcomes, social adjustment and quality of life were lacking in midpoint measurements compared with sleep and depression, and it is suggested that the reasons for the lack of detection at this stage be added in the paper.

5. Author mentioned”Growth models will be fitted to each variable separately and the correlations between the growth trajectories of the mediators and the outcome will be tested. ”It should be specified which indicators are used for evaluation and what the evaluation criteria are.

**Reviewer**	**2**
**Name**	**Siddiqui, Tahreem**
**Affiliation**	**Akershus University Hospital, Health Services Research Unit (HØKH)**
**Date**	**31-Jul-2024**
**COI**	**None**

Dear authors,

This is an interesting pilot trial. I have only a few comments written below regarding the method section.

1) Description of intervention: in the manuscript it is written that the therapist will have sessions weekly to “solve issues and answer questions." This is somewhat unclear what exactly the role of the therapist is and how that is linked to the online module; can you elaborate?

2) In the exclusion criteria, it is stated that “recent alterations of Psychopharmacotherapy (within last three months)” in Table 1, but using, for example, benzodiazepines or z-hypnotics might influence the intervention, as these drugs are sleep inducing or maintain sleep. I would recommend either specifying what drugs are in the exclusion criteria. Or discuss it briefly in the limitation section if applicable.

3) “These two will be measured once at pre-treatment, midpoint, post-treatment, and once at follow-up." What are the times of follow-ups? It is only written 3 months in Table 2, but is that the post-treatment or midpoint? Please clarify.

Good luck.

VERSION 1 - AUTHOR RESPONSE

Dear editor and reviewers, Thank you for allowing us the opportunity to revise our manuscript for the renewed consideration of your journal.

We have carefully read and revised according to your suggestions and comments. Attached you will find our responses point by point. Additionally, in the file upload section you will find a marked copy of all changes as well as the new main document with all revisions integrated.

Our best and warmest regards

Osame Salim, Markus Jansson-Fröjmark, Christina Sandlund & Annika Norell.

Reviewer: 1

Dr. Yanping Bao, Peking University

Comments to the Author:

Thank you for giving me this opportunity to review this manuscript. There are some concerns before publication:

- 1. Author mentioned ”Process and outcome measures will be assessed weekly across a 4-week intervention period and at a 3-month follow-up”. Therefore, the time setting of the intervention period and follow-up period and the reasons for such setting should be elaborated in the methods section of this paper.

  Thank you for allowing us to clarify this point. We have revised the “intervention” section under “methods and analysis” to provide further details on the time points and their background.

- 2. The purpose of this project is to evaluate the initial efficacy of PIPI for short - and long-term sleep. Please describe the basis and criteria of short - and long-term sleep.

  We thank the reviewer for their comment and recognize the ambiguity of these terms as we stated them. We have therefore revised our stated purpose to read "evaluate the preliminary efficacy of PI at post-treatment and 3 month follow-up" We hope this is an acceptable clarification for the journal.

- 3. For the scale or questionnaire mentioned in the paper, there should be an introduction to the items, scoring principles and other information.

  We thank the reviewer for this comment regarding the lack of information on the measures used. We have now revised the measures section with the suggested information.

- 4. In secondary outcomes, social adjustment and quality of life were lacking in midpoint measurements compared with sleep and depression, and it is suggested that the reasons

for the lack of detection at this stage be added in the paper.

Thank you for highlighting this issue. We have strived to minimize participant fatigue of measure completion while balancing our need for data collection, as per your recommendation we have added a comment in the manuscript under "secondary outcomes" describing this reason for not measuring WSAS and BBQ at midpoint

- 5. Author mentioned "Growth models will be fitted to each variable separately and the correlations between the growth trajectories of the mediators and the outcome will be tested. "It should be specified which indicators are used for evaluation and what the evaluation criteria are.

We appreciate the reviewer commenting on this issue. We have updated the data-analysis section with a new data analytic approach that we believe better suits the sample and provided the indicators and evaluation criteria for this analysis.

Reviewer: 2
Mrs. Tahreem Siddiqui, Akershus University Hospital
Comments to the Author:
Dear authors,

This is an interesting pilot trial. I have only a few comments written below regarding the method section.

- 1) Description of intervention: in the manuscript it is written that the therapist will have sessions weekly to "solve issues and answer questions." This is somewhat unclear what exactly the role of the therapist is and how that is linked to the online module; can you elaborate?

Dear reviewer, thank you for allowing us the opportunity to clarify this statement. We agree that it was somewhat unclear and have now revised the manuscript to

more clearly describe the extent and content of these short calls.

- 2) In the exclusion criteria, it is stated that "recent alterations of Psychopharmacotherapy (within last three months)" in Table 1, but using, for example, benzodiazepines or z-hypnotics might influence the intervention, as these drugs are sleep inducing or maintain sleep. I would recommend either specifying what drugs are in the exclusion criteria. Or discuss it briefly in the limitation section if applicable.

  We appreciate the reviewer's comment on this matter. We have revised our inclusion and exclusion criteria to clarify that concurrent psychopharmacological treatment is permitted as long as the dosage remains stable, meaning participants take their medication daily. However, the use of 'as-needed' hypnotics during the trial is now clearly stated as an exclusion criterion, as it could compromise the interpretation of the trial's results. These changes have been matched with trial pre-registration.

3) "These two will be measured once at pre-treatment, midpoint, post-treatment, and once at follow-up." What are the times of follow-ups? It is only written 3 months in Table 2, but is that the post-treatment or midpoint? Please clarify.

We thank the reviewer for highlighting this ambiguity in the text, we have now revised the measures section to only contain references to table 2 for timepoints of the measures. We hope that this will make the matter clearer.
Good luck.

VERSION 2 - REVIEW

**Reviewer**	**2**
**Name**	**Siddiqui, Tahreem**
**Affiliation**	**Akershus University Hospital, Health Services Research Unit (HØKH)**
**Date**	**31-Aug-2024**
**COI**	**None**

Dear authors,

Thank you for revising and clarifying the manuscript. Regarding the prescribed medications (z-hypnotics/Benzo) I would recommend mentioning in the discussion limitation section something about the prescribed medication use and its effect on the intervention.

VERSION 2 - AUTHOR RESPONSE

Dear editorial office, I have now reformatted the references according to your comments. They are in ascending order with square brackets [ ].

I am however unable to edit the authors profile to submit the Orchid ID in ScholarOne. The system does not allow me to do this. OrcidID for all authors are available in the title page of the manuscript. It is okay on our part of you are unable to add this to the manuscript.

Reviewer comment:

"*Dear authors, Thank you for revising and clarifying the manuscript. Regarding the prescribed medications (z-hypnotics/Benzo) I would recommend mentioning in the discussion limitation section something about the prescribed medication use and its effect on the intervention."

- We agree with the reviewer that this is a potential limitation and have now added a paragraph in the limitation section, discussing how this might affect the study results, we hope this will be satisfactory to the reviewer.
